# The influence of dietary crude protein concentrations, grain types and arginine:lysine ratios on the performance of broiler chickens

**DOI:** 10.1016/j.aninu.2023.05.007

**Published:** 2023-05-26

**Authors:** Shemil P. Macelline, Peter V. Chrystal, Chanon Inanan, Mehdi Toghyani, Peter H. Selle, Sonia Yun Liu

**Affiliations:** a School of Life and Environmental Sciences, Faculty of Science, The University of Sydney, Camperdown, 2006, NSW, Australia; b Poultry Research Foundation within The University of Sydne, Camden, 2570, NSW, Australia; c Complete Feed Solutions, Howick, 2145, New Zealand; d Sydney School of Veterinary Science, Faculty of Science, The University of Sydney, Camden, 2570, NSW, Australia

**Keywords:** Amino acid, Broiler chickens, Energy, Protein, Sorghum, Wheat

## Abstract

The objective of this study was to investigate the effects of dietary crude protein (CP) concentrations, grain types and arginine:lysine ratios on performance parameters of broiler chickens. The 2 × 2 × 2 factorial array of dietary treatments harnessed two CP concentrations (210 and 170 g/kg), two feed grains (wheat and sorghum), and two arginine:lysine ratios (104 and 110). Each dietary treatment was offered to 7 replicates of 14 birds per floor pen, a total of 784 off-sex male, Ross 308 broilers, from 14 to 35 d post-hatch. The dietary CP reduction compromised weight gain by 10.0% (2078 versus 2310 g/bird) as a main effect and FCR by 7.51% (1.474 versus 1.371), subject to an interaction. In a three-way interaction (*P* = 0.008), expanded arginine:lysine ratios improved FCR by 2.30% in 170 g/kg CP, sorghum-based diets but compromised FCR by 2.12% in corresponding wheat-based diets. Sorghum was the more suitable feed grain in reduced-CP diets as sorghum generated significant advantages in weight gain of 7.59% (2154 versus 2002 g/kg) and FCR of 6.94% (1.421 versus 1.527) in birds offered 170 g/kg CP diets. Both dietary CP and feed grain generated significant and divergent impacts in apparent ileal digestibility coefficients for the majority of 16 assessed amino acids. Dietary CP reductions increased non-bound amino acid inclusions (NBAA) in wheat-based diets (48.96 versus 9.80 g/kg) to a greater extent than sorghum-based diets (35.3 versus 9.50 g/kg) and increasing dietary NBAA inclusions were linearly associated with compromised weight gain (*r* = −0.834; *P* < 0.001) and FCR (*r* = 0.862; *P* < 0.001). Increasing ratios of free arginine to lysine plasma concentrations were linearly (*r* = −0.466; *P* = 0.004) related to improvements in FCR. The implications of the observed outcomes are discussed and possible explanations are advanced.

## 1 Introduction

The growing global demand for chicken-meat makes sustainable nutrient utilisation an imperative to guarantee future food security. The global per capita chicken-meat consumption was 14.9 kg in 2022, which represents approximately 44% of total meat consumption, but is projected to increase to 15.1 kg in 2029 (OECD, 2022). Dietary inputs of 471 g of crude protein (CP) and 30.78 MJ metabolisable energy (ME) are required to generate 1 kg of edible chicken-meat, based on performance objectives and nutrient specifications from one breeding company. Therefore, meeting dietary CP (Liu et al., 2021) and energy density (Gopinger et al., 2017) requirements with precision will enhance sustainable chicken-meat production.

Wheat and sorghum are the principal feed grains in Australian broiler diets with wheat being dominant. The feasibility of dietary CP reductions from 222 to 193 and 165 g/kg in either maize- or wheat-based diets for broiler chickens was compared in Chrystal et al. (2021) and maize was the more suitable feed grain in reduced-CP diets. The growth performance of birds offered 165 g/kg CP, wheat-based diets was seriously compromised, but the 193 g/kg CP wheat-based diets supported statistically comparable growth performance to the 222 g/kg CP diets. This suggests that CP could be reduced in wheat-based diets by approximately 30 g/kg without negatively influencing growth performance. Birds offered reduced-CP, wheat-based diets performed satisfactorily in Yin et al. (2020), but not in Greenhalgh et al. (2020) and Chrystal et al. (2021). Consequently, the shortfalls of wheat have been reviewed given its apparent inferiority to maize in the context of reduced-CP diets (Selle et al., 2022a). Wheat has higher protein content than maize and requires higher inclusions of non-bound (synthetic, crystalline) amino acids to meet specifications in reduced-CP diets. Wheat has a more rapid starch digestion rate than maize (Giuberti et al., 2012) and contains more soluble non-starch polysaccharides (Bach Knudsen, 1997). It is then relevant that sorghum is similar to maize in these respects and this also applies to amino acid profiles (Selle et al., 2022a). Thus, sorghum may be a more suitable feed grain than wheat in the context of reduced-CP diets and for this reason the two feed grains are compared in the present study.

Arginine is an essential amino acid in broiler diets and is involved in multiple physiological pathways in poultry (Castro and Kim, 2020). Unlike most animal species, arginine is essential in poultry and broiler chickens have a comparatively high dietary arginine requirement of 11.0 to 12.5 g/kg (Ball et al., 2007). The high requirement stems from high rates of protein deposition in chickens, the lack of endogenous synthesis, and metabolic interactions between arginine and lysine (Ball et al., 2007). An undesirable property of reduced-CP diets is increased lipid deposition as monitored by relative abdominal fat-pad weights. Over a series of three similar studies (Chrystal et al., 2020a, Chrystal et al., 2020b, Chrystal et al., 2020c) dietary CP reductions from 202 to 161 g/kg in maize-based diets increased relative fat-pad weights by an average of 71.4% (13.44 versus 7.84 g/kg). However, dietary arginine supplementation has been shown to decrease abdominal fat content in broiler chickens. Fouad et al. (2013) reported that the inclusion of 2.5 g/kg arginine in a maize-soy diet, containing 12.5 g/kg arginine and 11.1 g/kg lysine, significantly decreased fat-pad weights by 20.7% (15.7 versus 19.8 g/kg). Antagonistic arginine–lysine interactions are established in poultry (Austic and Scott, 1975); therefore, the dietary arginine to lysine ratio assumes importance. A dietary arginine to lysine ratio of 108 has been recommended (Wu, 2014a, Wu, 2014b), but there are indications that higher ratios may be advantageous (Zampiga et al., 2018; Castro et al., 2020; Corzo et al., 2021). Therefore, dietary arginine to lysine ratios of 104 and 110 were compared in the present study.

The present study was designed to compare wheat and sorghum as the feed grain basis of standard- and reduced-CP diets with two arginine:lysine ratios. Thus, the hypotheses tested were that sorghum is a more suitable feed grain than what in reduced-CP diets and that elevated arginine:lysine ratios will improve performance of broiler chickens offered reduced-CP diets.

## 2 Materials and methods

### 2.1 Animal ethics statement

This feeding study fully complied with the specific guidelines (2019/1651) approved by the Research Integrity and Ethics Administration of The University of Sydney.

### 2.2 Diet preparation

An outline of the dietary treatments is included in Table 1. The formulations of the experimental diets were based on near-infrared spectroscopy (NIR) of wheat, sorghum and soybean meal using the AMINOIR Advanced program (Evonik Nutrition & Care GmbH, Hanau, Germany). Sorghum and wheat were mediumly ground (4.0 mm hammer-mill screen) prior to being blended into complete diets which were steam-pelleted through a Palmer PP330 pellet press (Palmer Milling Engineering, Griffith, NSW, Australia) at a conditioning temperature of 80 °C with a conditioner residence time of 14 s and were then cooled. The composition and nutrient specifications of the experimental diets are shown in Table 2, Table 3, respectively. All diets contained exogenous phytase (Axtra PHY, Danisco Animal Nutrition) and xylanase (Axtra XB, Danisco Animal Nutrition) and 20 g/kg Celite (Celite Corporation, Lompoc, CA, USA) as an inert dietary marker. All diets were formulated to 11.0 g/kg digestible lysine, 13.1 g/kg glycine equivalents and DEB was maintained at 210 mEq/kg. The analysed starch, protein (N) and amino acid concentrations in the 8 dietary treatments are shown in Table 4. There are some discrepancies in the analysed amino acid concentrations, which, as mentioned below, were taken into account to calculate amino acid digestibility coefficients.

**Table 1:** Outline of 8 dietary treatments.

Experimental diet	Crude protein, g/kg	Feed grain	Arginine:lysine ratio
1A	210	Wheat	104
2B	210	Sorghum	104
3C	170	Wheat	104
4D	170	Sorghum	104
5E	210	Wheat	110
6F	210	Sorghum	110
7G	170	Wheat	110
8H	170	Sorghum	110

**Table 2:** Composition of experimental diets (g/kg, as-is basis).

Item	1A	2B	3C	4D	5E	6F	7G	8H
Sorghum	–	624	–	789	–	623	–	794
Wheat	674	–	877	–	679	–	863	–
Soybean meal	225	275	–	102	219	275	–	95.8
d,l-Methionine	2.68	3.21	4.31	4.66	2.72	3.21	4.38	4.72
Glycine	0.13	0.98	5.57	5.63	0.28	0.98	5.7	5.8
l-Arginine	0.69	0.56	6.77	5.52	1.53	1.23	7.51	6.37
l-Histidine	–	–	1.25	1.04	–	–	1.29	1.1
l-Isoleucine	0.44	0.13	3.82	2.82	0.54	0.14	3.89	2.92
l-Leucine	–	–	4.84	–	–	–	4.96	–
l-Lysine HCl	4.2	3.55	10.7	8.72	4.38	3.55	10.7	8.91
l-Phenylalanine	–	–	2.53	1.26	–	–	2.53	1.36
l-Threonine	1.68	1.35	4.53	3.6	1.76	1.35	4.58	3.68
l-Tryptophan	–	–	0.65	0.24	–	–	0.67	0.27
l-Tyrosine	–	–	2.14	0.59	–	–	2.26	0.69
l-Valine	0.9	0.5	4.3	3.17	1	0.5	4.38	3.27
Soy oil	43.9	45.1	12.3	19.6	43.1	45.1	17	18.7
Limestone	13.8	13.6	14.8	14.4	13.9	13.6	14.8	14.4
Monocalcium phosphorus	6.5	6.16	8.08	7.34	6.54	6.16	8.14	7.39
Potassium carbonate	–	–	6.44	2.62	–	–	6.51	2.85
Salt	1.38	3	–	–	1.19	3	–	–
Sodium bicarbonate	2.24	–	4.33	4.45	2.52	–	4.33	4.46
Vitamin-mineral premix^1^	2	2	2	2	2	2	2	2
Choline Cl (60%)	0.8	0.8	0.8	0.8	0.8	0.8	0.8	0.8
Feed enzymes^2^	0.2	0.2	0.2	0.2	0.2	0.2	0.2	0.2
Celite	20	20	20	20	20	20	20	20
Inert filler	–	–	2.37	–	–	–	10.8	–

1 The vitamin-mineral premix supplied per tonne of feed: retinol, 12,000 IU; cholecalciferol, 5000 IU; tocopheryl acetate, 75 mg; menadione, 3 mg; thiamine, 3 mg; riboflavin, 8 mg; niacin, 55 mg; pantothenate, 13 mg; pyridoxine, 5 mg; folate, 2 mg; cyanocobalamin, 16 μg; biotin, 200 μg; cereal-based carrier, 149 mg; Cu (sulphate), 16 mg; Fe (sulphate), 40 mg; I (iodide), 1.25 mg; Se (selenate), 0.3 mg; Mn (sulphate and oxide), 120 mg; Zn (sulphate and oxide), 100 mg; cereal-based carrier, 128 mg.

2 Phytase (Axtra PHY, Danisco Animal Nutrition) and xylanase (Axtra XB, Danisco Animal Nutrition).

**Table 3:** Nutrient specifications of experimental diets (g/kg).

Item	1A	2B	3C	4D	5E	6F	7G	8H
AME, MJ/kg	13.0	13.0	13.0	13.0	13.0	13.0	13.0	13.0
Crude protein	210	210	170	170	210	210	170	170
SID lysine	11.0	11.0	11.0	11.0	11.0	11.0	11.0	11.0
SID methionine	5.21	5.77	5.89	6.45	5.23	5.77	5.92	6.48
SID TSAA	8.14	8.14	8.14	8.14	8.14	8.14	8.14	8.14
SID threonine	7.26	7.26	7.26	7.26	7.26	7.26	7.26	7.26
SID valine	8.69	8.69	8.69	8.69	8.69	8.69	8.69	8.69
SID isoleucine	7.59	7.59	7.59	7.59	7.59	7.59	7.59	7.59
SID leucine	12.4	16.4	11.8	13.1	13.0	16.4	11.8	12.9
SID arginine	11.4	11.4	11.4	11.4	12.1	12.1	12.1	12.1
SID histidine	4.28	4.18	3.63	3.63	4.23	4.17	3.63	3.63
SID tryptophan	2.15	2.31	1.76	1.76	2.12	2.31	1.76	1.76
SID glycine equivalent 1	13.1	13.1	13.1	13.1	13.1	13.1	13.1	13.1
SID phenylalanine	8.54	8.96	4.84	6.06	8.43	8.96	4.76	5.95
SID Phenylalanine + tyrosine	13.5	14.8	11.6	11.6	13.4	14.8	11.6	11.6
Calcium	8.70	8.70	8.70	8.70	8.70	8.70	8.70	8.70
Available phosphorus	4.35	4.35	4.35	4.35	4.35	4.35	4.35	4.35
Crude fibre	20.4	20.4	17.6	18.2	20.4	20.4	17.3	18.1
Crude fat	61.5	72.5	30.5	50.4	60.7	72.5	34.7	49.5
DEB	210	210	210	210	210	210	210	210

SID = standard ileal digestibility; TSAA = total sulphur amino acids; DEB = dietary electrolyte balance = K^+^ + Na ^+^ – Cl^−^.

1 Glycine equivalent = glycine concentration + (serine concentration × 0.7143).

**Table 4:** Analysed nutrient composition of experimental diets (as fed-basis, g/kg).

Item	1A	2B	3C	4D	5E	6F	7G	8H
Dry matter	893	880	890	875	893	883	894	871
Crude protein	205	204	170	168	210	210	171	180
Starch	374	365	483	497	380	400	500	478
Arginine	11.5	11.7	10.9	10.9	12.7	11.5	11.7	12.0
Histidine	5.10	5.30	4.00	4.20	5.20	5.00	3.80	4.20
Isoleucine	8.40	9.20	7.40	8.30	8.90	8.90	7.40	8.60
Leucine	14.0	19.4	12.3	15.7	14.6	19.5	12.3	15.8
Lysine	11.6	12.1	11.1	10.9	12.5	11.3	11.1	11.6
Methionine	4.30	4.30	5.00	5.10	4.10	4.00	5.30	5.50
Phenylalanine	9.40	10.3	6.90	8.30	9.80	10.1	7.80	8.50
Threonine	8.10	8.50	7.40	7.60	8.50	8.10	7.40	7.90
Valine	9.80	10.3	9.20	9.50	10.3	10.0	9.30	9.80
Alanine	7.40	8.50	4.00	9.90	7.80	11.6	3.90	9.80
Aspartic acid	16.4	19.5	6.00	11.1	17.4	18.1	5.80	11.0
Glutamic acid	46.7	39.0	36.7	28.0	49.1	38.0	35.4	27.9
Glycine	8.20	8.50	10.2	9.50	8.70	8.10	10.7	10.1
Proline	14.3	12.1	13.0	9.50	14.8	12.2	11.8	9.70
Serine	9.30	9.60	5.50	6.30	9.70	9.40	5.40	6.40
Tyrosine	4.10	4.30	3.60	3.10	4.10	3.90	3.70	3.10
Total amino acids	189	196	153	158	198	190	153	162

### 2.3 Bird management

A total of 784 male Ross 308 one-day-old chicks were procured from a commercial hatchery and were initially offered a conventional starter diet (230 g/kg CP) from 1 to 13 d post-hatch. At d 14, birds were weighed and distributed to 56 floor pens to ensure an even body-weight distribution (average body-weight 491 ± 16.0 g/bird per pen). Each of the eight dietary treatments was offered to seven replicate pens (14 birds per pen) from 14 to 35 d post-hatch. The floor pen dimensions were 1.5 m in width and depth. Birds had unrestricted access to feed and water in an environmentally controlled facility under a lighting schedule of 18-h-on and 6-h-off. An initial room temperature of 32 °C was maintained for the first week, which was gradually decreased to 22 °C and kept constant to the end of the experiment.

### 2.4 Data and sample collection, chemical analyses, calculations

Growth performance (weight again, feed intake, FCR) was determined from 14 to 35 d post-hatch. Birds were weighed at d 14 and 35 and feed intakes were monitored over this interval, bodyweights of any dead or culled birds were recorded daily to correct feed intakes on a per pen basis and adjust FCR calculations.

At 34 d post-hatch, blood samples were taken from the brachial vein of three representative birds per pen to determine free amino acid concentrations in systemic plasma. Blood samples were centrifuged and decanted plasma samples were held at −80 °C prior to analysis. Concentrations of twenty proteinogenic amino acids were determined using precolumn derivatisation amino acid analysis with 6-aminoquinolyl-*N*-hydroxysuccinimidyl carbamate (AQC; Waters AccQTag Ultra; Waters Australia PL; www.waters.com) followed by separation of the derivatives and quantification by reversed phase ultra-performance liquid chromatography (RP-UPLC). All amino acids were detected by UV absorbance and this procedure is fully described in Selle et al. (2016).

At 35 d post-hatch, birds were euthanised by an intravenous injection of sodium pentobarbitone, abdominal cavities opened, and abdominal fat-pads dissected out and weighed and recorded against final body weights. The small intestine was removed and digesta was gently expressed manually from the distal half of the ileum and pooled by cage, homogenised, freeze dried and weighed to determine the apparent digestibility coefficients of starch, crude protein (N) and amino acids. Starch concentrations in diets and digesta samples were determined by using total starch assay kits (Megazyme, Wicklow, Ireland) as described in Mahasukhonthachat et al. (2010) and protein (N) concentrations were determined by methods described in Siriwan et al. (1993). Amino acid concentrations of diets and digesta were determined via 24 h liquid hydrolysis at 110 °C in 6 mol/L HCl followed by analysis of 16 amino acids using the Walters AccQTag Ultra chemistry on a Waters Acquity UPLC. Amino acid analyses were completed as outlined by Cohen and Michaud (1993). Apparent crude protein, starch and amino acid digestibility coefficients in distal jejunum and distal ileum were calculated by the following equation:

$$Digestibility\;coefficient=\frac{{\left(Nutrient/AIA\right)}_{Diet}-{\left(Nutrient/AIA\right)}_{Digesta}}{{\left(Nutrient/AIA\right)}_{Diet}}$$

Some discrepancies in analysed amino acid concentrations were detected, which mainly involved amino acids with high dietary inclusions as non-bound entities. Seven amino acids were included across all diets as both protein-bound and non-bound entities. Significant linear relationships between dietary non-bound amino acid inclusions and analysed concentrations of six amino acids were detected. These included four negative (isoleucine, lysine, threonine, valine) and two positive (methionine, glycine) relationships. These anomalies indicate that non-bound and protein-bound amino acids are not being extracted at identical rates during the analytical procedures. Therefore, calculations of apparent amino acid digestibility coefficients were adjusted by substituting total specified concentrations for the eleven amino acids that were included in experimental diets as non-bound entities for the analysed concentrations. Disappearance rates (g/bird per day) of protein (N), and starch in the distal ileum were calculated as the product of dietary concentrations of nutrient (g/kg), daily feed intake (g/day) from 14 to 35 d post-hatch and the relevant digestibility coefficient. Carcass yields were obtained from the manual processing of four birds selected at random from each pen. Breast and leg quarters were removed in their entirety, weighed and recorded against final body weights.

### 2.5 Statistical analysis

Experimental data were analysed as 2 × 2 × 2 factorial array by analyses of variance using the JMP Pro 16.0 software package (SAS Institute Inc. JMP Software. Cary, NC). The model used for the analyses of variance was as follows:

*Y*_*ijkt*_ = *μ* + *α*_*i*_ + *β*_*j*_ + *γ*_*k*_ + (*αβ*)_*ij*_ + (*αγ*)_*ik*_ + (*βγ*)_*jk*_ + (*αβγ*)_*ijk*_ + *ε*_*ijkt*._

where, *Y*: the *t*-th response observed for treatment *i*, *j*, *k*; *μ*: overall mean; *α*_*i*_: effect on the response due to the *i*th level of factor 1; *βj*: effect on the response due to the *j*th level of factor 2; *γ*_*k*_: effect on the response due to the *k*th level of factor 3; *ε*_*ijkt*_: independent random variables.

Two-way interactions: *αβ*, *αγ*, *βγ*. Three-way interaction: *αβγ*.

Linear and quadratic regressions and Pearson correlations were established when considered appropriate. Pen means were the experimental units and a probability level of less than 5% was considered statistically significant.

## 3 Results

The effects of dietary treatments on growth performance and relative abdominal fat pad-weights shown as Table 5. A feed grain by CP interaction (*P* = 0.013) was observed for weight gain because birds offered 170 g/kg CP sorghum-based diets had a 7.59% advantage (2154 versus 2002 g/bird) over their wheat-based counterparts. Reducing dietary CP depressed feed intake by 3.50% (3057 versus 3168 g/bird; *P* < 0.001). There was a three-way treatment interaction (*P* = 0.008) for FCR, where there were no statistical differences between birds offered 210 g/kg CP diets. However, sorghum-based diets supported an FCR of 1.421 in comparison to 1.527 for wheat-based diets in 170 g/kg CP diets. Moreover, expanded arginine:lysine ratios significantly improved FCR with sorghum-based diets by 2.30% (1.404 versus 1.437) but compromised FCR by 2.12% (1.543 versus 1.511) with wheat-based diets. A feed grain by CP interaction (*P* = 0.006) was observed for relative abdominal fat-pad weights. This was largely because birds offered 170 g/kg CP, sorghum-based diets had 40.1% heavier relative abdominal fat-pad weights (11.91 versus 8.50 g/kg) than their wheat-based counterparts.

**Table 5:** Effects of dietary treatments on growth performance and relative abdominal fat pad-weights from 14 to 35 d post-hatch.

Treatment			Growth performance			Relative fat-pad weights, g/kg
Crude protein, g/kg	Feed grain	Arginine:lysine ratio	Weight gain, g/kg	Feed intake, g/bird	FCR, g/g	
210	Wheat	104	2252^c^	3123	1.387^a^^b^	8.09^a^
		110	2308^c^^d^	3161	1.369^a^	8.65^a^^b^
	Sorghum	104	2332^d^	3192	1.369^a^	9.65^b^^c^
		110	2343^d^	3192	1.362^a^	10.15^c^
170	Wheat	104	2024^a^	3058	1.511^d^	8.55^a^^b^
		110	1979^a^	3053	1.543^d^	8.45^a^^b^
	Sorghum	104	2147^b^	3085	1.437^c^	12.15^d^
		110	2161^b^	3034	1.404^b^	11.67^d^
SEM			26.0	36.7	0.0097	0.043
Main effects: feed grain						
Wheat			2137	3098	1.455	8.45
Sorghum			2243	3128	1.394	10.95
CP, g/kg						
210			2310	3168^b^	1.371	9.15
170			2078	3057^a^	1.474	10.21
Arginine:lysine ratio						
104			2181	3111	1.423	9.66
110			2198	3110	1.420	9.73
Significance (*P-*value)						
FG			<0.001	0.302	<0.001	<0.001
CP			<0.001	<0.001	<0.001	0.002
Arginine:lysine ratio			0.623	0.868	0.345	0.710
Feed grain × CP			0.013	0.382	<0.001	0.006
Feed grain × arginine:lysine ratio			0.840	0.423	0.058	0.742
CP × arginine:lysine ratio			0.179	0.375	0.375	0.219
Feed grain × CP × Arginine:lysine ratio			0.168	0.936	0.008	0.807

CP = crude protein.

a b ^c d^ Within a column, means without a common superscript differ at *P* < 0.05.

As shown in Table 6, the dietary CP reduction decreased *Pectoralis major* yields by 6.22% (181 versus 193 g/kg) but did not significantly affect *Pectoralis minor* yields. The dietary CP reduction increased leg quarter yields by 2.61% (236 versus 230 g/kg; *P* = 0.021) and expanded arginine:lysine ratios slightly, but significantly decreased leg quarter yields.

**Table 6:** Effects of dietary treatments on relative weights (g/kg) of carcass traits at d 35 post-hatch.

Treatment			*Pectoralis major*	*Pectoralis minor*	Leg quarters
Crude protein, g/kg	Feed grain	Arginine:lysine ratio			
210	Wheat	104	204	34.7	223
		110	181	31.7	233
	Sorghum	104	193	31.8	229
		110	194	28.7	234
170	Wheat	104	187	31.3	232
		110	186	32.1	238
	Sorghum	104	179	30.5	234
		110	172	30.8	241
SEM			5.9	2.11	3.8
Main effects: feed grain					
Wheat			189	32.5	231
Sorghum			184	30.5	234
CP, g/kg					
210			193^b^	31.7	230^a^
170			181^a^	31.2	236^b^
Arginine:lysine ratio					
104			191	32.1	229^b^
110			183	30.8	227^a^
Significance (*P-*value)					
Feed grain			0.247	0.191	0.282
CP			0.007	0.715	0.021
Arginine:lysine ratio			0.087	0.402	0.012
Feed grain × CP			0.169	0.524	0.901
Feed grain × arginine:lysine ratio			0.295	0.928	0.698
CP × arginine:lysine ratio			0.389	0.237	0.844
Feed grain × CP × arginine:lysine ratio			0.105	0.954	0.454

CP = crude protein.

a b Within a column, means without a common superscript differ at *P* < 0.05.

Dietary treatment effects on protein and starch apparent ileal digestibility coefficients, disappearance rates and starch to protein disappearance rate ratios are shown in Table 7. Sorghum-based diets supported fractionally higher starch digestibility coefficients than wheat (0.996 versus 0.993; *P* = 0.034) and dietary CP reductions improved protein digestibility by 1.32% (0.845 versus 0.834; *P* = 0.042). A three-way treatment interaction (*P* < 0.001) was observed for starch disappearance rates because expanded arginine:lysine ratios in 210 g/kg CP, sorghum-based diets significantly increased disappearance rates by 11.4% (60.68 versus 54.49 g/bird per day). Dietary CP reductions depressed protein disappearance rates by 18.8% (21.20 versus 26.10 g/bird per day; *P* < 0.001) and expanded arginine:lysine ratios increased protein disappearance rates by 3.28% (23.92 versus 23.16 g/bird per day; *P* = 0.034). A three-way treatment interaction (*P* < 0.001) was observed for starch to protein disappearance rate ratios. Expanded arginine:lysine ratios in 210 g/kg CP, sorghum-based diets significantly increased disappearance rate ratios from 2.12 to 2.28 and increased disappearance rate ratios from 3.29 to 3.45 in 170 g/kg CP, wheat-based diets.

**Table 7:** Effects of dietary treatments on protein and starch digestibility coefficients, disappearance rates and starch to protein disappearance rate ratios in distal ileum at 35 d post-hatch.

Treatment			Digestibility coefficients		Disappearance rates, g/bird per day		Starch to protein disappearance rate ratios
Crude protein, g/kg	Feed grain	Arginine:lysine ratio	Starch	Protein	Starch	Protein	
210	Wheat	104	0.995	0.844	55.34^a^	25.72	2.15^a^
		110	0.995	0.837	56.92^a^	26.45	2.16^a^
	Sorghum	104	0.994	0.830	54.49^a^	25.75	2.12^a^
		110	0.997	0.826	60.68^b^	26.35	2.28^b^
170	Wheat	104	0.992	0.859	69.79^c^^d^	21.26	3.29^d^
		110	0.989	0.840	71.98^d^^e^	20.89	3.45^e^
	Sorghum	104	0.997	0.836	72.74^e^	20.64	3.53^e^
		110	0.995	0.846	69.01^c^	21.99	3.13^c^
SEM			0.0018	0.0075	0.880	0.367	0.029
Main effects: feed grain							
Wheat			0.993^a^	0.845	64.07	23.50	2.81
Sorghum			0.996^b^	0.835	63.96	23.61	2.75
CP, g/kg							
210			0.995	0.834^a^	58.86	26.10^b^	2.18
170			0.993	0.845^b^	71.19	21.20^a^	3.38
Arginine:lysine ratio							
104			0.995	0.843	63.72	23.16^a^	2.82
110			0.994	0.837	64.38	23.92^b^	2.74
Significance (*P-*value)							
Feed grain			0.034	0.060	0.259	0.702	0.884
CP			0.150	0.042	<0.001	<0.001	<0.001
Arginine:lysine ratio			0.693	0.326	0.018	0.034	0.523
Feed grain × CP			0.079	0.714	0.250	0.802	0.054
Feed grain × arginine:lysine ratio			0.580	0.159	0.607	0.137	<0.001
CP × arginine:lysine ratio			0.183	0.918	0.001	0.731	<0.001
Feed grain × CP × arginine:lysine ratio			0.838	0.235	<0.001	0.088	<0.001

CP = crude protein.

a b ^c d e^ Within a column, means without a common superscript differ at *P* < 0.05.

Dietary treatment effects on apparent ileal digestibility coefficients of essential amino acids are shown in Table 8. A feed grain by CP interaction (*P* = 0.001) was observed for leucine because in 170 g/kg CP, wheat-based diets supported superior leucine digestibility by 7.63% (0.889 versus 0.826) in comparison to sorghum-based diets. As a main effect, wheat generated significantly higher digestibility coefficients for histidine (6.83%), isoleucine (2.92%), phenylalanine (3.06%) and valine (2.30%) than sorghum, where the percentage increases are shown in parentheses. Alternatively, sorghum supported higher digestibility coefficients for arginine (1.33%) and lysine (1.81%). Reducing dietary CP levels significantly increased digestibilities of arginine (2.22%), isoleucine (5.54%), lysine (3.08%), methionine (0.95%), phenylalanine (2.71%), threonine (4.05%) and valine (4.78%). Increasing the dietary arginine:lysine ratio significantly depressed histidine and threonine digestibilities by 1.58% and 1.95%, respectively.

**Table 8:** Effects of dietary treatments on apparent digestibility coefficients of essential amino acids in distal ileum at 35 d post-hatch.

Treatment			Arginine	Histidine	Isoleucine	Leucine	Lysine	Methionine	Phenylalanine	Threonine	Valine
Crude protein, g/kg	Feed grain	Arginine:lysine ratio									
210	Wheat	104	0.896	0.842	0.843	0.845^b^	0.874	0.946	0.865	0.801	0.829
		110	0.892	0.831	0.839	0.837^a^^b^	0.869	0.949	0.859	0.794	0.822
	Sorghum	104	0.903	0.798	0.826	0.831^a^^b^	0.888	0.953	0.844	0.792	0.814
		110	0.904	0.788	0.811	0.817^a^	0.875	0.946	0.834	0.776	0.800
170	Wheat	104	0.918	0.865	0.897	0.897^c^	0.899	0.957	0.900	0.851	0.876
		110	0.905	0.840	0.881	0.881^c^	0.885	0.951	0.884	0.819	0.855
	Sorghum	104	0.925	0.792	0.865	0.830^ab^	0.915	0.960	0.859	0.827	0.847
		110	0.927	0.789	0.861	0.822^a^^b^	0.912	0.963	0.853	0.823	0.844
											
SEM			0.0049	0.062	0.0080	0.0092	0.0067	0.0037	0.0075	0.0087	0.0083
Main effects: feed grain											
Wheat			0.903^a^	0.845^b^	0.866^b^	0.866	0.882^a^	0.952	0.875^b^	0.817	0.846^b^
Sorghum			0.915^b^	0.791^a^	0.841^a^	0.825	0.898^b^	0.954	0.849^a^	0.805	0.827^a^
CP, g/kg											
210			0.899^a^	0.814	0.830^a^	0.832	0.876^a^	0.948^a^	0.850^a^	0.791^a^	0.816^a^
170			0.919^b^	0.822	0.876^b^	0.858	0.903^b^	0.957^b^	0.873^b^	0.823^b^	0.855^b^
Arginine:lysine ratio											
104			0.911	0.825^b^	0.860	0.852	0.895	0.954	0.864	0.819^b^	0.843
110			0.907	0.812^a^	0.848	0.839	0.885	0.952	0.860	0.803^a^	0.830
Significance (*P-*value)											
Feed grain			0.001	<0.001	<0.001	<0.001	0.002	0.348	<0.001	0.066	0.002
CP			<0.001	0.224	<0.001	<0.001	<0.001	0.001	<0.001	<0.001	<0.001
Arginine:lysine ratio			0.337	0.027	0.091	0.092	0.069	0.584	0.070	0.022	0.055
Feed grain × CP			0.518	0.097	0.759	0.001	0.233	0.078	0.218	0.717	0.874
Feed grain × arginine:lysine ratio			0.163	0.291	0.926	0.999	0.861	0.084	0.732	0.416	0.650
CP × arginine:lysine ratio			0.562	0.785	0.977	0.945	0.953	0.883	0.789	0.595	0.842
Feed grain × CP × arginine:lysine ratio			0.539	0.333	0.335	0.585	0.260	0.875	0.478	0.142	0.281

CP = crude protein.

a b ^c^ Within a column, means without a common superscript differ at *P* < 0.05.

The digestibility outcomes for non-essential amino acids are shown in Table 9. Feed grain by CP interactions were observed for alanine, aspartic acid and proline (*P* < 0.001) and also tyrosine (*P* = 0.013). A weak three-way treatment interaction (*P* = 0.024) was observed for serine. The strong interactions were driven by large differences in amino acid digestibilities between feed grains pursuant to the dietary CP reduction. In 170 g/kg CP diets, sorghum supported noticeably higher mean digestibilities for alanine (0.822 versus 0.678) and aspartic acid (0.783 versus 0.650); whereas, wheat supported higher digestibilities for proline (0.911 versus 0.754) and tyrosine (0.871 versus 0.778). As main effects, wheat-based diets generated higher glutamic acid digestibility by 8.50% (0.919 versus 0.847; *P* < 0.001) and the dietary CP reduction increased glycine digestibility by 9.92% (0.864 versus 0.786; *P* < 0.001).

**Table 9:** Effects of dietary treatments on apparent digestibility coefficients of non-essential amino acids in distal ileum at 35 d post-hatch.

Treatment			Alanine	Aspartic acid	Glutamic acid	Glycine	Proline	Serine	Tyrosine
Crude protein, g/kg	Feed grain	Arginine:lysine ratio							
210	Wheat	104	0.783^c^	0.783^c^	0.913	0.790	0.892^c^	0.824^d^^e^	0.839^d^^e^
		110	0.784^c^	0.787^c^	0.913	0.783	0.891^c^	0.826^e^	0.828^c^^d^
	Sorghum	104	0.823^c^	0.802^c^	0.858	0.791	0.787^b^	0.813^d^^e^	0.800^b^^c^
		110	0.801^c^	0.776^c^	0.845	0.780	0.782^b^	0.795^b^^c^^d^	0.758^a^
170	Wheat	104	0.704^b^	0.688^b^	0.931	0.878	0.923^d^	0.808^c^^d^^e^	0.878^f^
		110	0.651^a^	0.632^a^	0.919	0.853	0.899^c^	0.762^a^	0.863^e^^f^
	Sorghum	104	0.825^c^	0.784^c^	0.846	0.862	0.753^a^	0.778^a^^b^	0.780^a^^b^
		110	0.819^c^	0.782^c^	0.841	0.864	0.754^a^	0.782^a^^b^^c^	0.776^a^^b^
									
SEM			0.0158	0.0142	0.007	0.0082	0.0078	0.0106	0.0104
Main effects: feed grain									
Wheat			0.729	0.720	0.919^b^	0.827	0.902	0.804	0.852
Sorghum			0.817	0.785	0.847^a^	0.826	0.768	0.791	0.778
CP, g/kg									
210			0.797	0.786	0.882	0.786^a^	0.838	0.814	0.805
170			0.750	0.721	0.884	0.864^b^	0.833	0.782	0.824
Arginine:lysine ratio									
104			0.783	0.762	0.887	0.833	0.839	0.805	0.825
110			0.764	0.744	0.879	0.820	0.832	0.791	0.806
Significance (*P-*value)									
Feed grain			<0.001	<0.001	<0.001	0.803	<0.001	0.089	<0.001
CP			<0.001	<0.001	0.699	<0.001	0.344	<0.001	0.020
Arginine:lysine ratio			0.083	0.054	0.143	0.079	0.198	0.060	0.021
Feed grain × CP			<0.001	<0.001	0.051	0.893	<0.001	0.296	0.013
Feed grain × arginine:lysine ratio			0.605	0.562	0.799	0.318	0.364	0.329	0.505
CP × arginine:lysine ratio			0.398	0.389	0.815	0.864	0.460	0.407	0.245
Feed grain × CP × arginine:lysine ratio			0.132	0.045	0.324	0.188	0.204	0.024	0.160

CP = crude protein.

a b ^c d e f^ Within a column, means without a common superscript differ at *P* < 0.05.

The effects of dietary treatments on free plasma concentrations of essential and non-essential amino acids are shown in Table 10 and Table 11, respectively. Feed grain by CP interactions were observed for isoleucine (*P* = 0.034) and alanine (*P* = 0.048). With isoleucine, concentrations decreased from 17.8 to 15.0 μg/g following the dietary CP reductions in wheat-based diets, but increased from 11.6 to 14.0 μg/g in sorghum-based diets. Concentrations of alanine decreased markedly from 106.8 to 67.7 μg/g following the dietary CP reductions in wheat-based diets, but in sorghum-based diets the decrease was relatively modest, from 106.5 to 95.1 μg/g. Wheat-based diets generated significantly higher free plasma concentrations of histidine, valine, cysteine, glutamic acid, glutamine, glycine, proline and serine; whereas, sorghum-based diets generated higher concentrations of arginine, leucine and asparagine. Dietary CP reductions significantly increased concentrations of arginine (35.2%), lysine (60.2%), methionine (78.4%), threonine (79.2%), valine (15.0%) and glycine (59.0%), but decreased aspartic acid (24.5%) and cysteine (12.0%). Expanded arginine:lysine ratios significantly increased arginine concentrations by 31.6% but decreased tryptophan by 17.7%. Concentrations of phenylalanine and tyrosine were not statistically influenced by treatment.

**Table 10:** Effects of dietary treatments on essential amino acid plasma concentrations (μg/g) in broiler chickens.

Treatment			Arginine	Histidine	Isoleucine	Leucine	Lysine	Methionine	Phenylalanine	Threonine	Tryptophan	Valine
Crude protein, g/kg	Feed grain	Arginine:lysine ratio										
210	Wheat	104	39.5	8.1	19.3^b^	28.0	37.0	23.6	24.8	92.5	6.5	41.1
		110	53.2	7.6	16.2^a^^b^	23.6	35.2	21.4	20.3	72.1	5.3	35.6
	Sorghum	104	43.7	6.4	11.6^a^	31.5	42.6	19.0	21.5	61.4	6.7	24.0
		110	61.9	7.0	11.6^a^	32.6	33.9	19.0	23.3	66.7	5.4	24.5
170	Wheat	104	50.7	6.2	15.6^a^^b^	24.4	67.9	38.5	23.6	139.8	6.9	41.2
		110	63.9	5.2	14.3^a^	23.0	58.1	39.0	22.9	132.3	5.2	38.8
	Sorghum	104	68.7	5.4	14.2^a^	29.2	58.1	35.2	25.2	127.1	5.0	32.6
		110	87.6	5.3	13.8^a^	28.9	54.5	35.9	24.7	125.5	4.5	31.4
												
SEM			8.17	0.71	1.66	2.43	5.68	3.26	2.19	14.00	3.25	3.25
Main effects: feed grain												
Wheat			51.8^a^	6.7^b^	16.4	24.8^a^	49.6	30.6	22.9	109.2	5.9	39.2^b^
Sorghum			65.5^b^	6.0^a^	12.8	30.6^b^	47.3	27.3	23.7	95.2	5.4	28.1^a^
CP, g/kg												
210			50.0^a^	7.3	14.7	28.9	37.2^a^	20.8^a^	22.5	73.2^a^	5.9	31.3^a^
170			67.6^b^	5.5	14.5	26.4	59.6^b^	37.1^b^	24.1	131.2^b^	5.4	36.0^b^
Arginine:lysine ratio												
104			50.6^a^	6.5	15.2	28.3	51.4	29.1	23.8	105.2	6.2^b^	34.7
110			66.6^b^	6.3	14.0	27.0	45.4	28.9	22.8	99.2	5.1^a^	32.6
Significance (*P-*value)												
Feed grain			0.029	0.001	0.005	0.002	0.574	0.161	0.618	0.166	0.252	<0.001
CP			0.004	0.158	0.871	0.149	<0.001	<0.001	0.301	<0.001	0.254	0.049
Arginine:lysine ratio			0.010	0.614	0.317	0.473	0.147	0.935	0.535	0.547	0.023	0.352
Feed grain × CP			0.219	0.437	0.034	0.799	0.279	0.965	0.544	0.672	0.150	0.195
Feed grain × arginine:lysine ratio			0.657	0.516	0.401	0.349	0.967	0.788	0.287	0.429	0.567	0.427
CP × arginine:lysine ratio			0.995	0.357	0.782	0.817	0.861	0.745	0.819	0.881	0.836	0.878
Feed grain × CP × arginine:lysine ratio			0.952	0.901	0.632	0.545	0.423	0.808	0.333	0.620	0.467	0.611

CP = crude protein.

a b Within a column, means without a common superscript differ at *P* < 0.05.

**Table 11:** Effects of dietary treatments on non-essential amino acid plasma concentrations (μg/g) in broiler chickens.

Treatment			Alanine	Asparagine	Aspartic acid	Cysteine	Glutamic acid	Glutamine	Glycine	Proline	Serine	Tyrosine
Crude protein, g/kg	Feed grain	Arginine:lysine ratio										
210	Wheat	104	122.0^d^	37.5	17.3	19.6	31.9	411.0	72.2	87.8	81.1	59.0
		110	91.6^b^^c^	30.9	13.1	16.5	27.6	337.0	55.7	77.9	63.6	44.0
	Sorghum	104	106.9^c^^d^	33.5	15.8	13.4	24.0	257.0	58.0	50.6	62.2	55.5
		110	106.0^c^^d^	37.2	10.9	13.8	22.8	232.0	57.1	53.7	62.3	55.1
170	Wheat	104	71.8^a^^b^	18.7	9.3	15.3	23.8	375.0	98.4	72.9	73.0	51.6
		110	63.5^a^	20.6	8.4	15.3	25.4	360.0	109.0	74.6	75.6	51.5
	Sorghum	104	98.2^b^^c^^d^	29.6	12.6	12.0	23.8	298.0	93.5	48.6	61.7	54.3
		110	92.0^b^^c^	28.1	12.9	12.9	24.0	265.0	85.1	46.8	59.6	56.4
SEM			9.56	4.22	2.23	1.34	2.13	31.70	7.21	7.28	7.03	5.47
Main effects: feed grain												
Wheat			87.2	26.9^a^	12.0	16.6^b^	27.2^b^	371^b^	83.6^b^	78.3^b^	73.3^b^	51.5
Sorghum			100.8	32.1^b^	13.1	13.0^a^	23.7^a^	263^a^	73.4^a^	49.9^a^	61.4^a^	55.3
CP, g/kg												
210			106.3	34.8	14.3^b^	15.8^b^	26.6	309	60.7^a^	67.5	67.3	53.4
170			81.4	24.3	10.8^a^	13.9^a^	24.3	324	96.5^b^	60.7	67.4	53.5
Arginine:lysine ratio												
104			99.7	29.8	13.8	15.1	25.9	335.0	80.5	65.0	69.5	55.1
110			88.3	29.2	11.3	14.6	25.0	299.0	76.7	63.3	65.2	51.8
Significance (*P-*value)												
Feed grain			0.054	0.001	0.515	0.001	0.025	<0.001	0.050	<0.001	0.023	0.327
CP			0.001	0.093	0.034	0.047	0.133	0.502	<0.001	0.194	0.968	0.987
Arginine:lysine ratio			0.100	0.837	0.134	0.646	0.555	0.113	0.457	0.740	0.397	0.350
Feed grain × CP			0.048	0.190	0.081	0.400	0.071	0.345	0.437	0.656	0.722	0.993
Feed grain × arginine:lysine ratio			0.253	0.781	0.942	0.253	0.775	0.732	0.870	0.650	0.378	0.282
CP × arginine:lysine ratio			0.539	0.559	0.183	0.347	0.240	0.565	0.342	0.746	0.523	0.270
Feed grain × CP × arginine:lysine ratio			0.321	0.258	0.785	0.491	0.446	0.454	0.100	0.428	0.272	0.424

CP = crude protein.

a b ^c d^ Within a column, means without a common superscript differ at *P* < 0.05.

## 4 Discussion

Overall growth performance in the present study was highly satisfactory as Ross 308 performance objectives (Aviagen, 2019) for weight gain were exceeded by 18.6% (2193 versus 1849 g/bird) and for FCR by 9.99% (1.423 versus 1.581). This is despite that reducing dietary CP by 40 g/kg compromised weight gain by 10.9% (2078 versus 2310 g/bird) and FCR by 7.51% (1.474 versus 1.371) as main effects. However, the dietary CP reduction depressed weight gain by 7.87% (2154 versus 2338 g/bird) and FCR by 4.03% (1.421 versus 1.366) in birds offered sorghum-based diets when average dietary arginine:lysine ratios are combined. In contrast, growth performance of birds offered wheat-based diets was compromised to greater extents with marked depressions of 12.2% (2002 versus 2280 g/bird) in weight gain and 11.5% (1.527 versus 1.378) in FCR. These data both reflect the challenges to the successful development of reduced-CP diets and indicate that sorghum is a more suitable feed grain than wheat in this context.

Instructively, NBAA inclusions were comparable (wheat: 9.80 g/kg, sorghum: 9.50 g/kg) in 210 g/kg CP diets, but in 170 g/kg CP diets NBAA inclusions were considerably higher in wheat-based diets (48.96 versus 35.33 g/kg). The higher NBAA inclusions in wheat-based diets are driven by the higher protein content of wheat (139 g/kg) than sorghum (107 g/kg) used in the present study, which is a typical difference. However, Baker (2009) contended that there are limits to the extent that intact protein can be replaced by non-bound amino acids to achieve maximal weight gain and feed efficiency. If so, wheat is disadvantaged relative to sorghum in the framework of reduced-CP broiler diets. Moreover, it may be deduced that increasing dietary NBAA inclusions were linearly associated with less efficient weight gain (*r* = −0.834; *P* < 0.001) and FCR (*r* = 0.862; *P* < 0.001) in the present study. While not conclusive, these relationships are consistent with the proposal that NBAA inclusions can become excessive in reduced-CP diets.

Intestinal uptakes of NBAA are more rapid than their protein-bound counterparts (Wu, 2009) and the implication is that non-bound and protein-bound amino acids are not bioequivalent (Selle et al., 2022b). The likelihood is that this difference promotes post-enteral imbalances between non-bound and protein-bound amino acids leading to post-prandial oxidation of surplus amino acids (Selle et al., 2022b). For example, non-bound leucine was more susceptible to post-prandial oxidation than protein-bound leucine in rats (Nolles et al., 2009). The catabolism of surplus amino acids is an obvious loss, but it is accompanied by an ‘energy cost’ because an energy input of 60.7 kJ is required to eliminate 1 g of uric acid-N generated by amino acid catabolism (Van Milgen, 2021).

A three-way interaction (*P* = 0.008) between CP, feed grain and arginine:lysine ratio was observed for FCR in the present study. In birds offered 170 g/kg CP sorghum-based, expanding arginine:lysine ratios improved FCR by 2.30%, but depressed FCR by 2.12% in their wheat-based counterparts. Arginine and lysine requirements for broiler chickens were determined by Nogueira et al. (2022) and in male birds, optimal ratios ranged from 107 to 118 depending on age. However, increasing dietary arginine:lysine ratios from 88 to 113 in maize-based diets improved FCR by 4.91% (1.55 versus 1.63) in Castro et al. (2020). This parallels the response in sorghum-based diets in the present study, but not wheat-based diets. In something of a precedent, elevated BCAA inclusions in 187.5 g/kg CP, wheat-based diets significantly depressed FCR by 8.33% (1.665 versus 1.537), but fractionally improved FCR in sorghum-based diets (1.378 versus 1.390) in Greenhalgh et al., 2022a, Greenhalgh et al., 2022b. Moreover, elevated BCAA inclusions decreased weight gain by 9.49% (1288 versus 1423 g/bird) in wheat-based diets, but increased gain by 9.26% (1451 versus 1328 g/bird) in sorghum-based diets in this study. Concentrations of non-bound BCAA, especially leucine, were substantially higher in wheat-than sorghum-based diets in Greenhalgh et al., 2022a, Greenhalgh et al., 2022b and these imbalances may have contributed to the observed responses. In the present study, wheat-based diets contained more non-bound arginine (16.50 versus 13.68 g/kg) and lysine (23.28 versus 19.29 g/kg) than sorghum and, reciprocally, sorghum-based diets contained more protein-bound amino acids. These differences may have exacerbated the recognised antagonism between arginine and lysine (Balnave and Brake, 2002); the likely basis of this antagonism is that a relative excess of lysine may impede the renal re-absorption of arginine (Maynard and Kidd, 2022). This may have contributed to the treatment interaction observed for FCR in the present study.

The importance of considering starch and protein digestive dynamics in tandem was evident in the present study as condensing ileal starch to protein disappearance rate ratios were quadratically associated with improvements in weight gain (*r* = 0.805; *P* < 0.001) and FCR (*r* = 0.780; *P* < 0.001). The positive impacts of capping dietary starch:protein ratios and, in turn, condensing starch to protein disappearance rate ratios on growth performance of birds offered reduced-CP, wheat- and maize-based diets has been previously reported (Greenhalgh et al., 2020, 2022b). Dietary starch:protein ratios will typically expand in the formulation of reduced-CP diets and any strategies that will limit this trend should be advantageous.

Perturbations in apparent amino acid digestibilities pursuant to dietary CP reductions are commonly observed (Liu et al., 2021) and constitute an impediment to the precise formulation of reduced-CP diets to meet amino acid requirements. The genesis of these perturbations is the opposing forces that are in play. Average digestibilities of five amino acids (Ala, Asp, Glu, Pro, Ser) that were present only as protein-bound entities in the present experiment decreased by 4.56% (0.690 versus 0.723) following the reduction from 210 to 170 g/kg CP. In contrast, average digestibilities of seven amino acids (arginine, isoleucine, lysine, methionine, threonine, valine, glycine) that were included as non-bound entities across all diets increased by 4.12% (0.885 versus 0.850). Dietary CP reductions can reduce apparent amino acid digestibility coefficients because concentrations of dietary amino acids in distal ileal digesta are diluted by amino acids derived from endogenous secretions and the gut microbiota. This shift in amino acid concentrations depresses apparent digestibility coefficients (Donkoh and Moughan, 1994). In addition, there are variations in inherent amino acid digestibilities of the three key feedstuffs: soybean meal, wheat and sorghum. Ravindran et al. (1999) reported that the mean ileal digestibility of 14 amino acids in soybean meal was 0.816 in comparison to 0.774 for wheat and 0.743 for sorghum. Therefore, the partial substitution of soybean meal with either feed grain in the formulation of reduced-CP diets will tend to depress amino acid digestibilities. Interestingly, histidine digestibility in wheat was superior to sorghum by 12.4% (0.782 versus 0.696) in Ravindran et al. (1999), which was reflected in the present study as wheat generated higher histidine digestibilities than sorghum by 6.83% (0.845 versus 0.791) as a main effect. Theoretically, NBAA are completely digestible (Lemme et al., 2005), which will counteract the above two negative factors when amino acids are included in diet formulations as non-bound entities at high inclusion levels. For example, the reduction in dietary CP increased lysine digestibility by 3.08% (0.903 versus 0.876; *P* < 0.001) where lysine-HCl inclusions ranged from 3.55 to 10.70 g/kg in the present study.

**Fig. 1:**
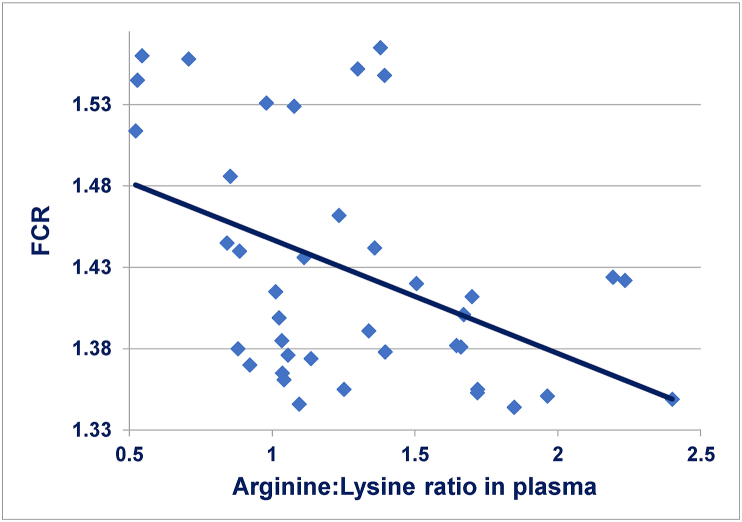
Negative linear relationship (*r* = −0.446; *P* = 0.004): *y* = 1.517 – 0.070 *x*, where *y* is FCR and *x* is the ratio of arginine to lysine plasma concentrations.

Free amino acid concentrations in systemic plasma are difficult to interpret because they reflect the dynamic equilibrium between post-enteral amino acid availability and protein accretion, which is complicated by protein degradation, catabolism and gluconeogenesis involving amino acids (Fernández-Fígares et al., 1997). Dietary CP reductions significantly increased free plasma concentrations of methionine, lysine, threonine, valine, arginine; these increases could be indicative of inefficient utilisation of these pivotal amino acids. Also, plasma concentrations of methionine (*r* = 0.625; *P* < 0.001), glycine (*r* = 0.674; *P* < 0.001), lysine (*r* = 0.584; *P* < 0.001), and threonine (*r* = 0.569; *P* < 0.001) were linearly related to increases in FCR or compromised feed efficiency. Again, these positive relationships could be indicative of inefficient amino acid utilisation for protein deposition. However, elevated plasma free threonine concentrations are frequently observed in broiler chickens following dietary CP reductions and could even serve as a biomarker for the adequacy with which reduced-CP diets are formulated (Macelline et al., 2021). Instructively, there is a negative linear relationship (*r* = −0.446; *P* = 0.004) between the ratio of free arginine to lysine plasma concentrations and FCR, as shown in Fig. 1. Thus, increases in arginine relative to lysine in the systemic circulation was associated with enhanced FCR and the linear equation predicts that an increase in plasma ratios from 1.0 to 2.0 would enhance FCR by 4.84% (1.377 versus 1.447). This may reflect post-enteral antagonistic interactions between arginine and lysine antagonism (Kadirvel and Kratzer, 1974).

## 5 Conclusion

It was established that sorghum is a more suitable feed grain than wheat in reduced-CP broiler diets as there was a CP × feed grain interaction (*P* = 0.013); sorghum supported a 2.41% greater increase in weight gain than wheat in 210 g/kg CP diets, but this advantage expanded to 7.59% in 170 g/kg CP diets. Increasing dietary arginine:lysine ratios per se did not influence growth performance, but a three-way FCR interaction (*P* = 0.008) showed that increasing arginine:lysine ratios in 170 g/kg CP, sorghum-based diets generated a 2.30% improvement in FCR as opposed to a 2.12% depression in FCR in corresponding wheat-based diets.

## Author contributions

All six authors contributed towards the completion of this study and have read and approved this manuscript. **Sonia Yun Liu** was the principal investigator of the relevant project and is the corresponding author. **Peter Vincent Chrystal** formulated the diets. **Shemil Priyan Macelline** and **Mehdi Toghyani** conducted and supervised the feeding study. **Peter Henry Selle** and **Shemil Priyan Macelline** completed the statistical analyses. **Chanon Inanan** completed the initial manuscript, which was completed by **Peter Henry Selle** and **Shemil Priyan Macelline** and **Sonia Yun Liu** was responsible for the final editing and submission of the manuscript.

## Declaration of competing interest

We declare that we have no financial and personal relationships with other people or organizations that can inappropriately influence our work, and there is no professional or other personal interest of any nature or kind in any product, service and/or company that could be construed as influencing the content of this paper.
